# Qualitative assessments of myocardial ischemia by cardiac MRI and coronary stenosis by invasive coronary angiography in relation to quantitative perfusion by positron emission tomography in patients with known or suspected stable coronary artery disease

**DOI:** 10.1007/s12350-018-01555-1

**Published:** 2018-12-10

**Authors:** Shahnaz Akil, Fredrik Hedeer, Marcus Carlsson, Håkan Arheden, Jenny Oddstig, Cecilia Hindorf, Jonas Jögi, David Erlinge, Henrik Engblom

**Affiliations:** 1 Department of Clinical Sciences Lund, Clinical Physiology, Skane University Hospital, Lund University, 22185 Lund, Sweden; 2 Department of Radiological Sciences, College of Health and Rehabilitation Sciences, Princess Nourah bint Abdulrahman University, Riyadh, Saudi Arabia; 3 Radiation Physics, Skåne University Hospital, Lund, Sweden; 4 Department of Clinical Sciences Lund, Department of Cardiology, Skane University Hospital, Lund University, Lund, Sweden

**Keywords:** Cardiac magnetic resonance imaging, cardiac positron emission tomography, coronary angiography, coronary artery disease, myocardial flow reserve

## Abstract

**Background:**

To relate findings of qualitative evaluation of first-pass perfusion-CMR and anatomical evaluation on coronary angiography (CA) to the reference standard of quantitative perfusion, cardiac PET, in patients with suspected or known stable coronary artery disease (CAD).

**Methods and Results:**

Forty-one patients referred for CA due to suspected stable CAD, prospectively performed adenosine stress/rest first-pass perfusion-CMR as well as ^13^N-NH_3_ PET on the same day, 4 ± 3 weeks before CA. Angiographers were blinded to PET and CMR results. Regional myocardial flow reserve (MFR) < 2.0 on PET was considered pathological. Vessel territories with stress-induced ischemia by CMR or vessels with stenosis needing revascularization had a significantly lower MFR compared to those with no regional stress-induced ischemia or vessels not needing revascularization (*P* < 0.001). In 4 of 123 vessel territories with stress-induced ischemia by CMR, PET showed a normal MFR. In addition, 12 of 123 vessels that underwent intervention showed normal MFR assessed by PET.

**Conclusion:**

The limited performance of qualitative assessment of presence of stable CAD with CMR and CA, when related to quantitative ^13^N-NH_3_ cardiac PET, shows the need for fully quantitative assessment of myocardial perfusion and the use of invasive flow reserve measurements for CA, to confirm the need of elective revascularization.

**Electronic supplementary material:**

The online version of this article (10.1007/s12350-018-01555-1) contains supplementary material, which is available to authorized users.

## Introduction

Coronary artery disease (CAD) is the leading cause of mortality worldwide. Therefore, accurate methods for diagnosing CAD are desirable. In the case of stable CAD, myocardial perfusion (MP) is affected predominantly during stress due to one or several flow-limiting stenoses, in the coronary arteries, resulting in stress-induced ischemia.

In patients with suspected stable CAD, assessment of presence and severity of stress-induced ischemia is of importance for prognosis.^1^–^3^ In addition, current guidelines from the European Society of Cardiology (ESC) and the American Heart Association (AHA) recommend the evaluation of stress-induced ischemia non-invasively, in patients with intermediate risk of stable CAD, before taking decisions regarding revascularization.^1^,^4^,^5^ Fractional flow reserve (FFR) assessment of intermediate stenoses found on coronary angiography (CA) is also recommended before an intervention since coronary angiography alone has been shown to have low diagnostic accuracy compared to FFR.^6^ Despite this knowledge and the current recommendations, coronary interventions are still performed in many patients based on the presence of anatomically significant stenosis on the CA, clinical status and risk factors^7^ without any prior assessment of stress-induced ischemia.^8^,^9^

First-pass cardiac magnetic resonance imaging (CMR) is one of the non-invasive methods available for the assessment of stress-induced ischemia. In clinical routine and in large clinical studies, qualitative visual assessment of the regional MP distribution is usually performed.^10^–^13^ Semi-quantitative methods with CMR are not as widely used, while the fully quantitative approaches are still being developed and validated.^14^–^16^

Dynamic cardiac positron emission tomography (PET) is another non-invasive method and considered the reference method for assessment of stress-induced ischemia because of its ability to quantify regional and global MP (ml/min/g) at rest and stress and thereby myocardial flow reserve (MFR).

Therefore, the aim of this study was to relate findings of qualitative evaluation of first-pass perfusion-CMR and clinical assessment of coronary angiography (CA) to quantitative perfusion assessed by cardiac PET in patients with suspected or known stable coronary artery disease (CAD).

## Methods

### Study Population

A total of 41 patients (9 females, mean age 67 years ± 7, age range 50-86 years) with suspected or known stable CAD, clinically referred for CA in conjunction with possible elective percutaneous coronary intervention (PCI), were prospectively included from November, 2013 to December, 2016. Patients were referred for CA in conjunction with possible PCI due to experience of stable angina symptoms in addition to clinical history wih risk factors in the majority of patients (80%) and due to results of prior non-invasive stress-testing in the minority of patients (20%). Patients with atrial fibrillation, known chronically occluded vessels, claustrophobia, asthma, severe chronic obstructive lung disease, and glomerular filtration rate < 30 ml/min were excluded. The patients performed stress/rest first-pass perfusion-CMR (1.5 T) as well as ^13^N-NH_3_ cardiac PET on the same day (4-5 hour apart), with the same sequence in all patients, at mean 4 ± 3 weeks (0.5-14 weeks) before CA. The CMR and cardiac PET examinations were performed for the purpose of the present study and kept blinded to the referring physician and the angiographer. The study was approved by the Regional Ethical Committee, Lund, Sweden and a written informed consent was obtained from all patients.

### Cardiac PET Imaging and Reconstruction

All patients performed the rest imaging first, followed by stress imaging approximately 1 hour later. At rest, patients were injected with ^13^N-NH_3_ at a constant flow rate. For the stress examination, ^13^N-NH_3_ was injected, at the same flow rate, 3 minutes after the beginning of adenosine infusion (140 μg/kg/min). Adenosine infusion continued, with continuous ECG monitoring, for another 4 minutes after isotope injection since the data from the first 4 minutes after isotope injection are used for calculation of the quantitative myocardial perfusion (see below). The injected activity of ^13^N-NH_3_ was 525 ± 78 MBq at both rest and stress.

For positioning, a scout view over the chest was performed followed by a low-dose computed tomography for attenuation correction (120 kV; 10 mAs, 10; rotation time 0.5 second). The PET acquisition was started simultaneously with the isotope injection, for both rest and stress examinations. Images were acquired using a GE Discovery 690 PET/CT with an acquisition time of 15 minutes for both stress and rest PET image acquisition.

Before reconstructing the images, evidence for patient motion was checked between the CT and PET images and manual adjustments were made. To ensure correction for the increase in accumulation of the metabolic product (^13^N-glutamine) trapped in the myocardial tissue, only the first 4 min of the PET acquisition were used, as recommended by DeGrado et al.^17^ to reconstruct the rest and stress dynamic images into 15 time-frames (12 × 10 s, 2 × 30 s and 1 × 60 s) using OSEM (3 iterations, 12 subsets) and a 5 mm post-filter.

### Cardiac PET Image Analysis

The reconstructed dynamic images were analyzed using the software Carimas (version 2.7, Turku, Finland). The left ventricle was delineated automatically with manual adjustments when needed. The activity in the blood and the myocardial wall as a function of time served as input information to the DeGrado compartment model^17^ for ^13^N-NH_3_, allowing quantification of the rest and stress global MP in ml/min/g as well as the regional MP in each of the three vessel territories (LAD, LCX, RCA). The regional MFR was calculated by dividing the stress by the rest regional MP. MFR < 2.0 was considered pathologic as previously suggested.^18^,^19^

### CMR Imaging and Reconstruction

Patients underwent perfusion-CMR in either a 1.5T Philips Achieva (Best, The Netherlands; n = 11) or Siemens, Magnetom Aera (Erlangen, Germany; n = 30) as the CMR scanner was replaced at the hospital during the study.

Three short-axis slices (basal, mid-ventricular, and apical) were acquired at rest and after 3 minutes of adenosine infusion (140 μg/kg/min), during the first-pass of 0.05 mmol/kg bolus of the gadolinium-based contrast agent Dotarem (Guerbet, Roissy, France). For the Philips scanner spatial resolution was 2 × 2 × 10 mm reconstructed to 1.4 × 1.4 × 10 mm and accelerating factor 3. For the Siemens scanner spatial resolution was 1.9 × 2.4 × 8 mm reconstructed to 1.9 × 1.9 × 8 mm and accelerating factor 3. Late gadolinium enhancement imaging (LGE) was performed to evaluate presence of infarction and fibrosis.

### CMR Image Analysis

All CMR analysis was performed using the software Segment version 2.0 (http://segment.heiberg.se)^20^ All stress/rest first-pass perfusion images were visually assessed for presence of stress-induced ischemia, by an experienced physician (HE) who was blinded to cardiac PET and CA results. A second experienced observer (MC), who was blinded to the results by the first experienced observer (HE) as well as to cardiac PET and CA results, re-assessed the first-pass CMR images of 14/41 patients which were randomly selected from the included population. A hypo-enhanced area at stress, present during at least three heart cycles, but not present at rest and not corresponding to contrast enhancement on LGE, indicated stress-induced ischemia. Stress-induced ischemia was assigned to one of the three vessel territories (LAD, LCX, and RCA). LGE images were qualitatively assessed for presence of infarct or fibrosis. Scar size was determined from the LGE short-axis images using a recently described automated algorithm for scar quantification taking signal intensity distribution, coronary vessel territory, and partial volume effects into consideration.^21^

### Coronary Angiography

Angiographers were blinded to cardiac PET and CMR, which were strictly research-initiated examinations not asked for by the clinician. This design of the study allowed for assessment of how well clinical routine, often to a large extent dependent on assessing angiograms, can guide accurate treatment decisions. The angiographers performed the CA according to clinical routine (fractional flow reserve measurements performed only at the angiographer’s request). Angiograms were visually assessed by the cardiologist responsible for writing the clinical angiography report, not aware of any of the PET or CMR results. The angiograms were assessed according to the clinical routine taking available clinical data and patient history into account. Thus, no specific percentage of stenosis was considered significant, just the judgment of the angiographer performing the angiography, with or without iFR/FFR. Instantaneous wave-free ratio (iFR) > 0.89 and FFR > 0.80 were considered normal, as previously suggested.^22^,^23^ Flow reserve measurements (iFR or FFR) were performed in 17/123 vessels (14 iFR and 3 FFR) belonging to 15/41 patients.

### Statistical Analysis

Results are given in mean ± SD. A non-paired *t* test was used for assessment of differences in regional MFR by PET between normal vessels and vessels requiring revascularization by CA as well as between normal vessel territories and territories with stress-induced ischemia by first-pass CMR. All graphs were generated with the software Graphpad Prism version 6.07 (Graph Pad Software, Inc., La Jolla, CA, USA). Results with *P* values < 0.05 were considered statistically significant.

## Results

Characteristics of the included patients can be found in Table 1 and characteristics of the electively revascularized patients (n = 24) are shown in Table 2. Twenty-four of the 41 patients were revascularized, 20 by PCI and 4 by coronary artery bypass graft surgery. The CABG patients were not analyzed separate from the PCI patients. History of previous PCI was found in 11/24 revascularized patients and in 4/17 non-revascularized patients. The majority of the electively revascularized patients had single-vessel disease (19/24). Infarct by LGE was found in 15/41 patients (6 LAD, 8 RCA, 6 LCX) with a scar size of 9 ± 6%. Septal fibrosis of non-ischemic origin was detected in 1/41 patients. In the subset of first-pass CMR images which were assessed by two experienced observers (14/41), the assessments corresponded to each other in 100% of the cases regarding presence or absence of stress-induced ischemia. The hemodynamic response (blood-pressure and heart rate response) during stress CMR and cardiac PET is presented in Table 3. MP at rest did not exceed 1.4 ml/min/g for any patient.

**Table 1:** Baseline characteristics of the included patients (n = 41)

Age	67 ± 7	
Females	9	22%
BMI (kg/m^2^)	27 ± 3	
Smoker	5	12%
Previous smoker	21	51%
Prior PCI	17	41%
Prior CABG	3	7%
Prior myocardial infarction	11	27%
Diabetes	9	22%
Hypertension	29	71%
Hypercholesterolemia	27	66%
Heredity for coronary artery disease	9	22%
Betablockers	23	56%
ACE-inhibitor/ARB	19	46%
Statins	36	88%
Anti-coagulants	35	85%

*BMI*, body mass index; *PCI*, percutaneous coronary intervention; *CABG*, coronary artery bypass graft; *ACE*, angiotensin converting enzyme, *ARB*, angiotensin II receptor blockers; *LGE*, late gadolinium enhancement

**Table 2:** Characteristics by coronary angiography of the electively revascularized patients (n = 24)

1-vessel	19	79%
2-vessels	3	13%
3-vessels	2	8%
LAD	15	63%
LCX	7	29%
RCA	9	38%

**Table 3:** Hemodynamic response during CMR and PET

	At rest			Adenosine		
	CMR	Cardiac PET	*P*-value	CMR	Cardiac PET	*P*-value
Heart rate (beats per minute)*	68 ± 9	66 ± 7	0.5	82 ± 11	83 ± 10	0.8
Systolic pressure (mmHg)	138 ± 17	149 ± 17	0.004	134 ± 27	135 ± 19	0.7
Diastolic pressure (mmHg)	80 ± 12	80 ± 16	0.7	76 ± 16	0**	NA

*Mean of the average heart rate during adenosine

for each patient

**Not measured during adenosine cardiac PET due to use of doppler instead of blood-pressure cuff

### CMR in Relation to Cardiac PET

**Figure 1:**
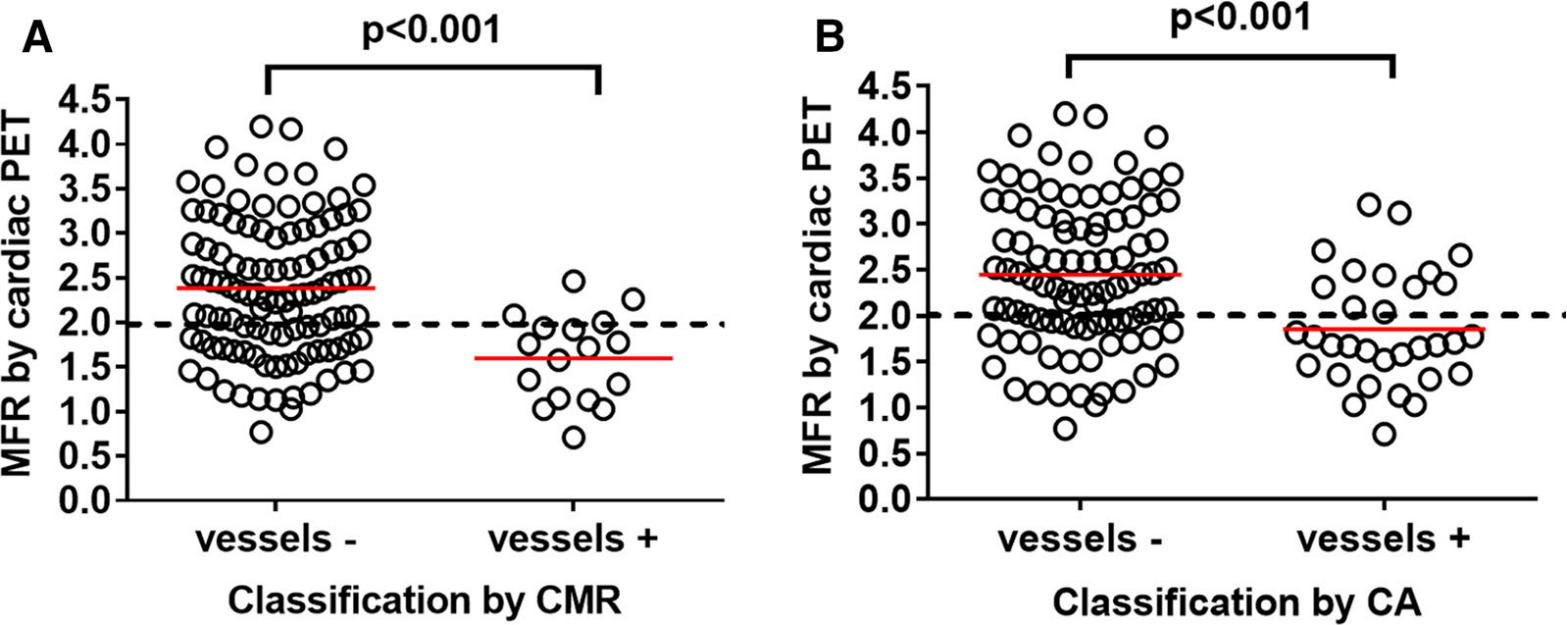
Myocardial flow reserve (MFR) by cardiac positron emission tomography (PET) for a total of 123 coronary arteries (41 patients X 3) classified by (**A**) cardiac magnetic resonance imaging (CMR) as normal (vessels -) or supplying territories with stress-induced myocardial ischemia (vessels +) (**B**) coronary angiography (CA) as normal/not needing revascularization (vessels -) or with stenosis that was electively revascularized (vessels +). Red lines indicate mean MFR and the dashed line indicates MFR cut-off at 2.0

There was significantly lower MFR in vessel territories with stress-induced myocardial ischemia by CMR (1.6 ± 0.5) compared to those with no regional stress-induced ischemia (2.4 ± 0.8) (*P *< 0.001, Figure 1A). CMR had a sensitivity of 47% and a specificity of 75% on a per-patient basis, and a sensitivity of 27% and a specificity of 96% on a per-vessel basis for MFR < 2.0 by PET (Table 4). Thus, there were several vessel territories (37/123, 30%) with decreased MFR (< 2.0) but normal first-pass perfusion by CMR. Furthermore, three vessel territories with stress-induced ischemia by first-pass perfusion-CMR had MFR > 2.0 (2.3 ± 0.2, range 2.1-2.5, Figure 1A). The experienced CMR observer reported low image quality in the images belonging to two of these vessel territories.

**Table 4:** Diagnostic accuracy of CA versus CMR, on a per-patient and per-vessel or -vessel territory analysis, using MFR by cardiac PET as reference

	Per-patient		Per-vessel or -vessel territory	
	CA (%)	CMR (%)	CA (%)	CMR (%)
Sensitivity	76	47	39	27
Specificity	54	75	83	96
PPV	54	57	63	82
NPV	76	67	66	65

### CA in Relation to Cardiac PET

There was significantly lower MFR in vessel territories supplied by revascularized arteries (1.9 ± 0.6) compared to non-revascularized arteries (2.4 ± 0.8) (*P *< 0.001, Figure 1B). CA had a sensitivity of 76% and a specificity of 54% on a per-patient basis, and a sensitivity of 39% and a specificity of 83% on a per-vessel basis, for MFR < 2.0 by PET (Table 4). Thus, there were several vessel territories with decreased MFR (< 2.0) but arteries evaluated by the angiographer as not needing revascularization (31/123, 25%). Furthermore, several vessels (12/123, 10%) that underwent intervention, after being evaluated by the angiographer as needing revascularization, had MFR > 2.0 (2.5 ± 0.4, range 2.04-3.0, Figure 1B). No invasive fractional flow reserve measurements were performed on these vessels.

### Invasive CA Measurements in Relation to Cardiac PET and CMR

In 16 of the 17 vessel territories where iFR or FFR were used, the iFR/FFR findings corresponded to the MFR findings by PET. A normal iFR/FFR was found in 12/12 vessel territories with MFR > 2.0. A decreased iFR/FFR was found in 4/5 vessel territories with MFR < 2.0. In one vessel territory with MFR < 2.0 (MFR = 1.5), iFR was normal (iFR = 1.0).

In 12 of the 17 vessel territories where iFR or FFR were used, the iFR/FFR findings corresponded to qualitative assessment by CMR. A normal iFR/FFR was found in 11/16 vessel territories with normal CMR. A decreased iFR/FFR was found in 1/1 vessel territory with pathologic CMR.

### Qualitative CMR and CA Versus Quantitative Cardiac PET

Two of the three vessel territories with stress-induced ischemia by CMR but MFR > 2.0 (2.3 ± 0.19, range 2.1-2.5) were supplied by vessels which underwent revascularization due to CA findings.

**Figure 2:**
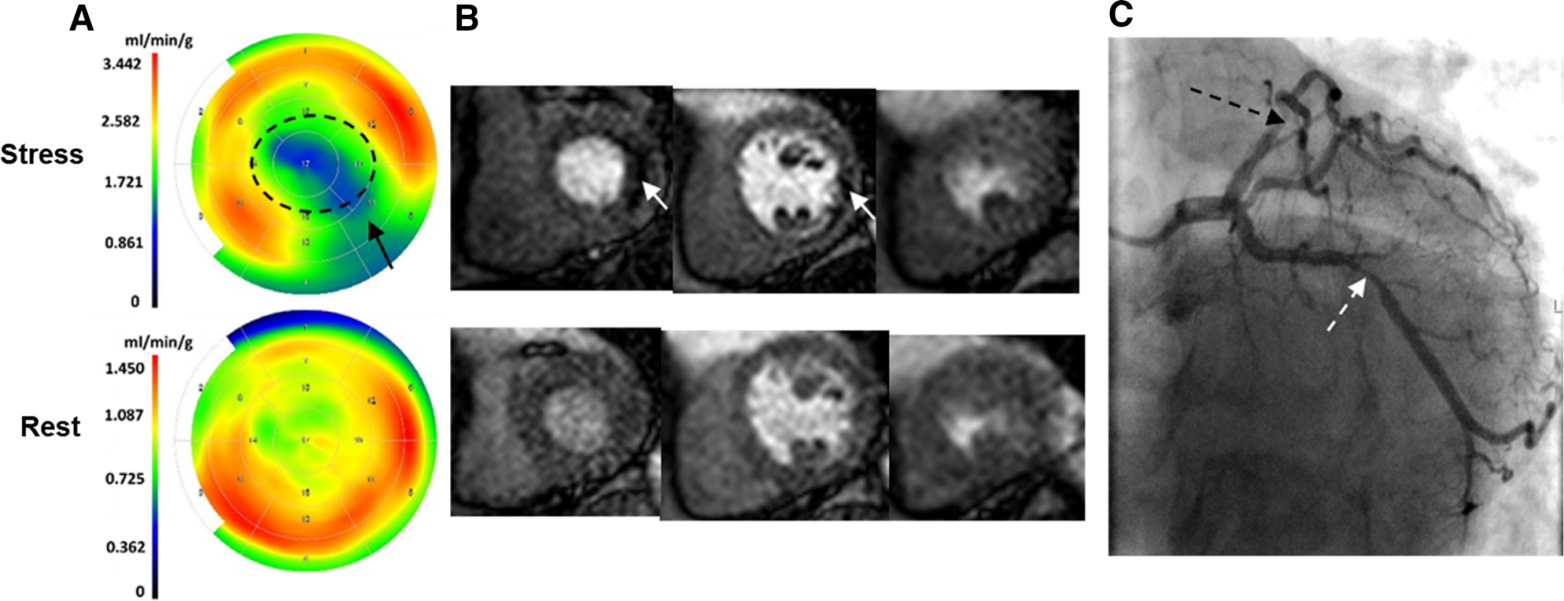
An example of a patient with a cardiac positron emission tomography (**A**) revealing abnormal myocardial blood flow at stress in both the LCX (black arrow) and LAD vessel territories (dashed circle). The bull’s eye plots represent the distribution of the quantified absolute myocardial blood flows (ml/min/g tissue) in the left ventricle. The color scales to the left of the bull’s eyes represent the flow ranges with warm colors indicating higher flows and cold colors lower flows. In this case first-pass perfusion-CMR (**B**) showed stress-induced ischemia only in the LCX territory (white arrows) while the angiogram (**C**) revealed a stenosis, evaluated as needing revascularization, in the LAD (dashed black arrow), not seen by CMR, in addition to a stenosis evaluated as needing revascularization in LCX (dashed white arrow)

Figure 2 shows an example of a patient where findings from CMR, CA and quantitative cardiac PET did not correspond to each other. The cardiac PET rest/stress bull’s eye plots show that the patient has 2-vessel disease (LCX, LAD), while the qualitative assessment of the first-pass CMR images reveal stress-induced ischemia in one vessel territory (LCX). The qualitative assessment of the CA revealed a stenosis evaluated as needing revascularization in the vessels supplying these territories with abnormal myocardial blood flow at stress by PET.

## Discussion

The present prospective study shows that the diagnostic accuracy of qualitative perfusion assessment with first-pass CMR or assessment with CA is limited in patients with suspected stable CAD, when considering quantitative cardiac ^13^N-NH_3_ PET as the reference standard. Given the significant number of patients and vessel territories with discrepancies between CMR or CA and MFR, there is a need for improvement in selection of patients with suspected CAD that should undergo elective revascularization. Furthermore, given that MFR also assesses microvascular disease, requiring a different treatment than epicardial coronary disease, there is a need for further studies of the myocardial pathophysiology underlying a MFR < 2.0 to improve prognosis and quality of life in this group of patients.

### CMR in Relation to Cardiac PET

In this study, the diagnostic accuracy for CMR in evaluating presence or absence of stable CAD, on both the per-patient and per-vessel analysis, was limited (Table 4). Previous studies have reported higher diagnostic accuracies for CMR, especially sensitivity and NPV, for the per-patient analysis^10^,^12^,^13^,^24^ when comparing to angiography. However, the majority of previously recorded diagnostic accuracies are based on a larger number of patients, different evaluation criteria for CMR and CA and a different study design, including ≥ 50% diameter stenosis on CA being the reference standard, as in the retrospective study by Jaarsma et al. Furthermore, for one previous study,^10^ the diagnostic accuracy of CMR in detecting angiographically defined CAD was based on presence of stress-induced ischemia on first-pass perfusion or presence of infarction/fibrosis on LGE or both.

Several vessel territories with decreased MFR, in the present study, had normal first-pass perfusion (37/123, 30%, Figure 1A). A possible explanation might be presence of left ventricular dysfunction, multi-vessel disease, or vascular disease not related to coronary stenosis, i.e. microvascular disease, and not affecting the relative in-flow kinetics in first-pass perfusion imaging. However, qualitative assessment by CMR has still been shown to be superior to other non-invasive imaging modalities (i.e. myocardial perfusion single photon emission tomography).^6^,^10^

In the current study, three of the vessel territories with normal MFR were classified as pathologic by CMR (Figures 1A and 2). This might be explained by poor image quality on CMR (which was reported in one case) or dark rim artifacts shown to affect the diagnostic accuracy of first-pass perfusion-CMR.^25^

### CA in Relation to Cardiac PET

In the present study, the diagnostic accuracy for CA in evaluating presence or absence of stable CAD, on both the per-patient and per-vessel analysis, was limited (Table 4). This is in line with the previously reported limited diagnostic accuracy of qualitative CA,^6^ but with a lower specificity for the per-patient analysis and a lower sensitivity for the per-vessel analysis found in the current study. However, in contrast to previous studies,^6^ a clinical decision of elective revascularization, and not a specific cut-off value of % diameter stenosis, was considered a positive finding in the current study.

Several vessels (n = 12) that underwent intervention, after being evaluated as having stenosis needing revascularization, had a normal MFR (Figure 1B), suggesting unnecessary revascularization of these vessels. In these vessels, no flow reserve measurements during CA were performed. The results in the current study, where evaluation of FFR or iFR was performed only in a minority of the patients (15/41, 37%), are thus in concordance with findings from a recent meta-analysis which concluded that qualitative CA had a lower diagnostic performance than other imaging modalities when related to invasive FFR measurements.^6^ The current study protocol included CA performed according to clinical routine, which did not require the angiographers to perform flow reserve measurements. For vessels where the angiographer decided to perform flow measurements during CA (n = 17), findings of MFR by cardiac PET corresponded to those of iFR/FFR in 16/17 vessel territories. Thus, the findings in the present study further emphasize what has previously been shown about the importance of the functional significance of a stenosis,^26^ and further encourage the use of flow reserve measurements during CA in patients with suspected significant stenosis.

Moreover, only 2/12 vessels, which had normal MFR but underwent intervention, due to presence of stenosis evaluated as needing revascularization by CA, showed stress-induced ischemia by CMR in the territories they supplied. This further highlights the need of using non-invasive stress-testing in patients with suspected CAD, to decrease the number of unnecessary CA and revascularizations, as recommended by the ESC and AHA guidelines.^4^ Furthermore, in the current study, several vessel territories (19/50, 38%, Figure 1B) with pathologic MFR had coronary stenosis not needing revascularization. Presence of microvascular disease can be a possible explanation for decreased MFR not detected by qualitative CA.

### MFR by Cardiac PET

The cut-off value of < 2.0 for ^13^N-NH_3_ cardiac PET, for a pathologic MFR, has been previously used in several studies.^18^,^19^,^27^ A previous study by Stuijfzand et al.^28^ has suggested that the use of the absolute quantification of MP at stress (in ml/min/g), instead of MFR, has similar diagnostic accuracy based on dynamic cardiac PET with [^15^O]H_2_O. However, there is a lack of studies investigating the diagnostic accuracy of cut-off values of absolute quantification of MP at stress (in ml/min/g) against a wider range of cut-off values of degree of stenosis by CA, including a population with suspected microvascular obstruction. Moreover, to the best of our knowledge, no cut-off value for absolute quantification of MP at stress using the radioisotope ^13^N-NH_3_ is available in the published literature. Thus, cut-off values for quantification in ml/min/g using ^13^N-NH_3_, as well as for other cardiac PET isotopes, are still debated and therefore we used MFR rather than absolute perfusion. MFR calculated from the regional stress and rest MP has been shown to be a sensitive measure to detect stable CAD and is valuable in the prediction of patient prognosis.^18^,^19^,^29^

Despite its quantitative ability, dynamic cardiac PET is not a widely used examination for the detection of stable CAD. The challenging logistics of the examination, which includes isotopes with short half-lives requiring the presence of an on-site cyclotron or isotope generator, make this examination costly.^30^

### Limitations

The following limitations should be considered when interpreting the results in this study. (1) The binary MFR cut-off value of 2.0 has its limitations when used to assess physiological processes such as MP. However, this cut-off is clinically established and has been used in several previous studies.^18^,^19^,^27^ Furthermore, it is known that MFR could be decreased because of non- epicardial CAD, such as microvascular disease, which CA and first-pass CMR cannot detect and which revascularization cannot treat. This limitation in using MFR as a reference could partly explain the low sensitivity found for both first-pass CMR and CA. Therefore, in the clinical routine quantitative and qualitative assessment of the myocardial perfusion should be combined for more accurate diagnosis. (2) The patients were included based on having a clinical referral for a CA with possible PCI, which means that the patients included in this study probably have a higher pre-test likelihood of CAD. Thus, these results can not be applied to patients with suspected CAD in general. (3) The use of CMR scanners from two different vendors might affect the qualitative assessment of first-pass CMR images due to difference in acquired spatial and temporal resolutions. (4) The majority of the revascularized patients had a stenosis evaluated as needing revascularization in the LAD (63%) and most of the revascularized patients (79%) had single-vessel disease by CA, which makes the generalization of the findings in the study somewhat limited for other coronary vessels and patients with multi-vessel disease. (5) The results might not be extrapolated to other PET isotopes such as ^82^Rb-PET for assessment of perfusion. (6) Adenosine stress imaging reflects the relative perfusion distribution within the major coronary vessel territories and usually not true ischemia, even though myocardium may become ischemic in cases of severe coronary stenosis due to proximal steel phenomenon. (7) The limited number of vessels (n = 17) where iFR/FFR was performed and the absence of quantitative CMR data limits the ability to make general conclusions on the relation between invasive flow measurements, CMR and MFR by cardiac PET.

## New Knowledge Gained

The present paper emphasizes the need for quantitative non-invasive assessment of myocardial perfusion as well as for increased use of invasive flow reserve measurements during coronary angiography, in patients with suspected stable CAD.

## Conclusion

The limited performance of qualitative assessment of the presence of stable CAD with CMR and CA, when related to quantitative ^13^N-NH_3_ cardiac PET, shows the need for fully quantitative assessment of myocardial perfusion and the use of invasive flow reserve measurements for CA, to confirm the need of elective revascularization in patients with suspected CAD.

## Electronic supplementary material

Below is the link to the electronic supplementary material.

Supplementary material 1 (PPTX 1352 kb)

## Abbreviations

AHA: American Heart Association
CAD: Coronary Artery Disease
CA: Coronary Angiography
CABG: Coronary artery bypass grafting
CMR: Cardiac magnetic resonance imaging
ESC: European Society of Cardiology
FFR: Fractional flow reserve
MFR: Myocardial flow reserve
MP: Myocardial Perfusion
PET: Positron Emission Tomography
PCI: Percutaneous coronary intervention
