# Supplementary material for: Qualitative assessments of myocardial ischemia by cardiac MRI and coronary stenosis by invasive coronary angiography in relation to quantitative perfusion by positron emission tomography in patients with known or suspected stable coronary artery disease
Source: J Nucl Cardiol. 2018 Dec 10;27(6):2351–9. doi: 10.1007/s12350-018-01555-1 (PMC7749089; doi:10.1007/s12350-018-01555-1)
Supplement: Supplementary file 1 — Supplementary material 1 (PPTX 1352 kb) [file 12350_2018_1555_MOESM1_ESM.pptx]

## Slide 1
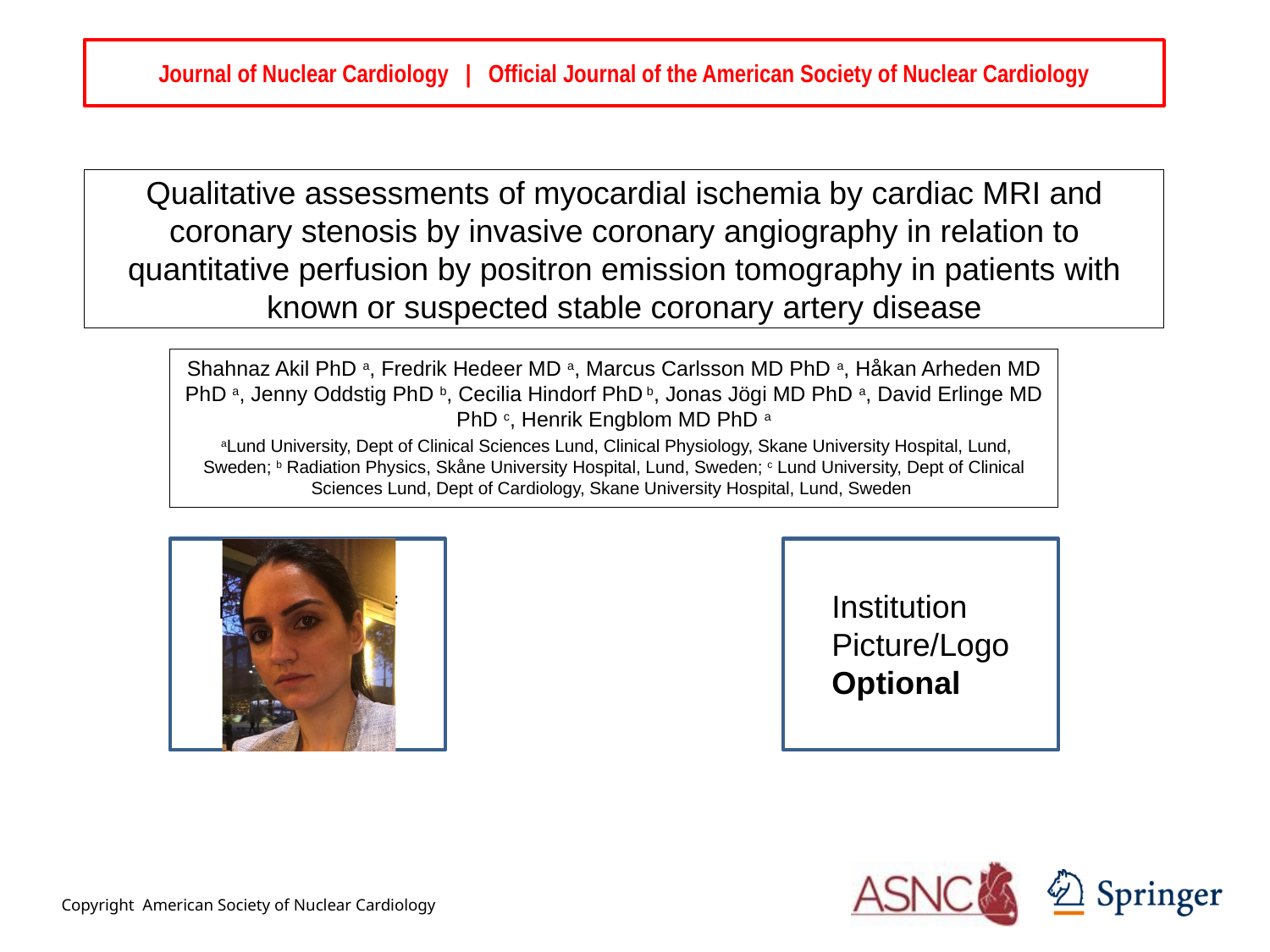

Journal of Nuclear Cardiology | Official Journal of the American Society of Nuclear Cardiology
# Qualitative assessments of myocardial ischemia by cardiac MRI and coronary stenosis by invasive coronary angiography in relation to quantitative perfusion by positron emission tomography in patients with known or suspected stable coronary artery disease
Shahnaz Akil PhD a, Fredrik Hedeer MD a, Marcus Carlsson MD PhD a, Håkan Arheden MD PhD a, Jenny Oddstig PhD b, Cecilia Hindorf PhD b, Jonas Jögi MD PhD a, David Erlinge MD PhD c, Henrik Engblom MD PhD a
 aLund University, Dept of Clinical Sciences Lund, Clinical Physiology, Skane University Hospital, Lund, Sweden; b Radiation Physics, Skåne University Hospital, Lund, Sweden; c Lund University, Dept of Clinical Sciences Lund, Dept of Cardiology, Skane University Hospital, Lund, Sweden
Head shot of author
required
Institution
Picture/Logo
Optional
Copyright American Society of Nuclear Cardiology

## Slide 2
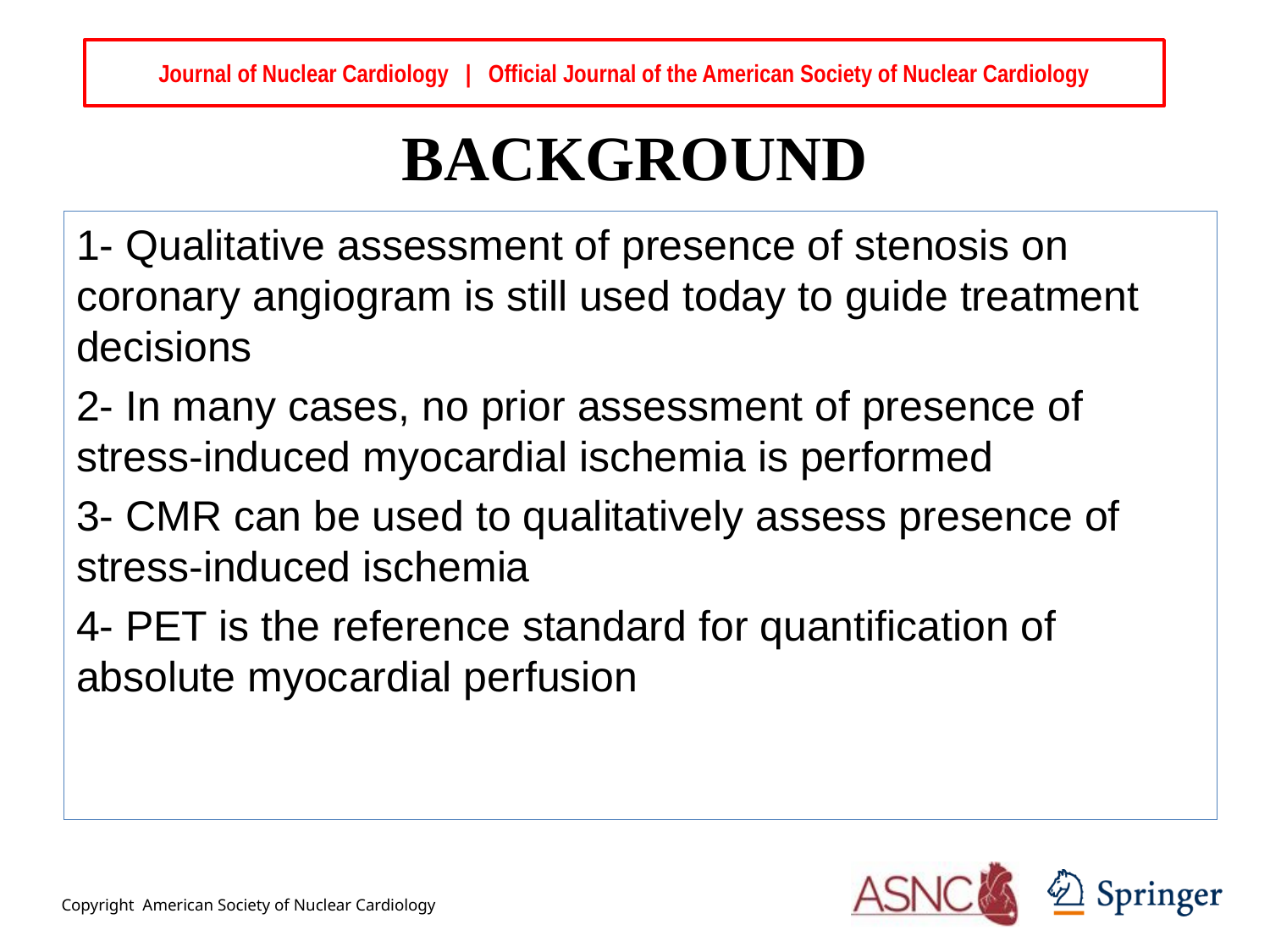

Journal of Nuclear Cardiology | Official Journal of the American Society of Nuclear Cardiology
# BACKGROUND
1- Qualitative assessment of presence of stenosis on coronary angiogram is still used today to guide treatment decisions
2- In many cases, no prior assessment of presence of stress-induced myocardial ischemia is performed
3- CMR can be used to qualitatively assess presence of stress-induced ischemia
4- PET is the reference standard for quantification of absolute myocardial perfusion
Copyright American Society of Nuclear Cardiology

## Slide 3
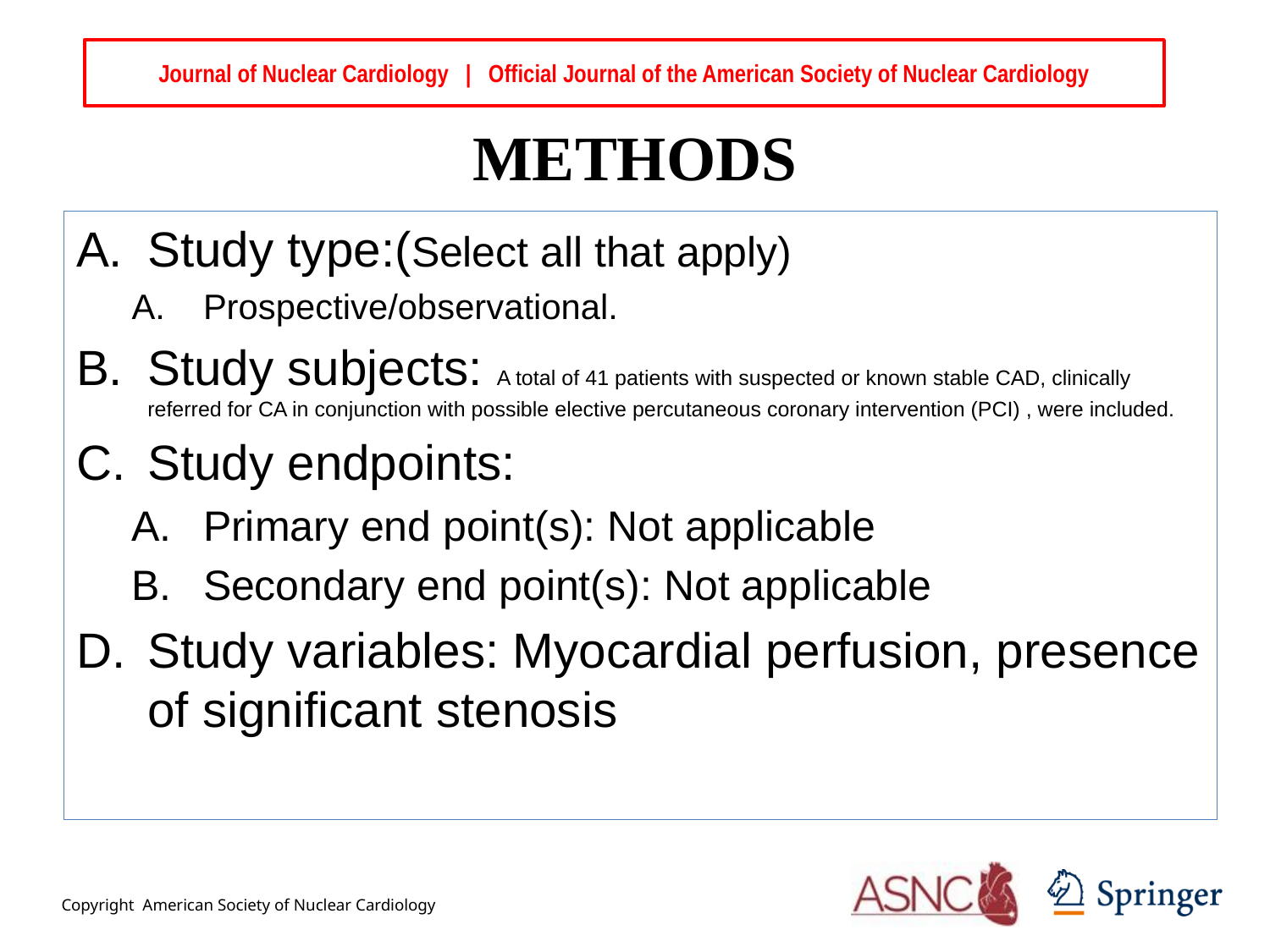

Journal of Nuclear Cardiology | Official Journal of the American Society of Nuclear Cardiology
# METHODS
Study type:(Select all that apply)
Prospective/observational.
Study subjects: A total of 41 patients with suspected or known stable CAD, clinically referred for CA in conjunction with possible elective percutaneous coronary intervention (PCI) , were included.
Study endpoints:
Primary end point(s): Not applicable
Secondary end point(s): Not applicable
Study variables: Myocardial perfusion, presence of significant stenosis
Copyright American Society of Nuclear Cardiology

## Slide 4
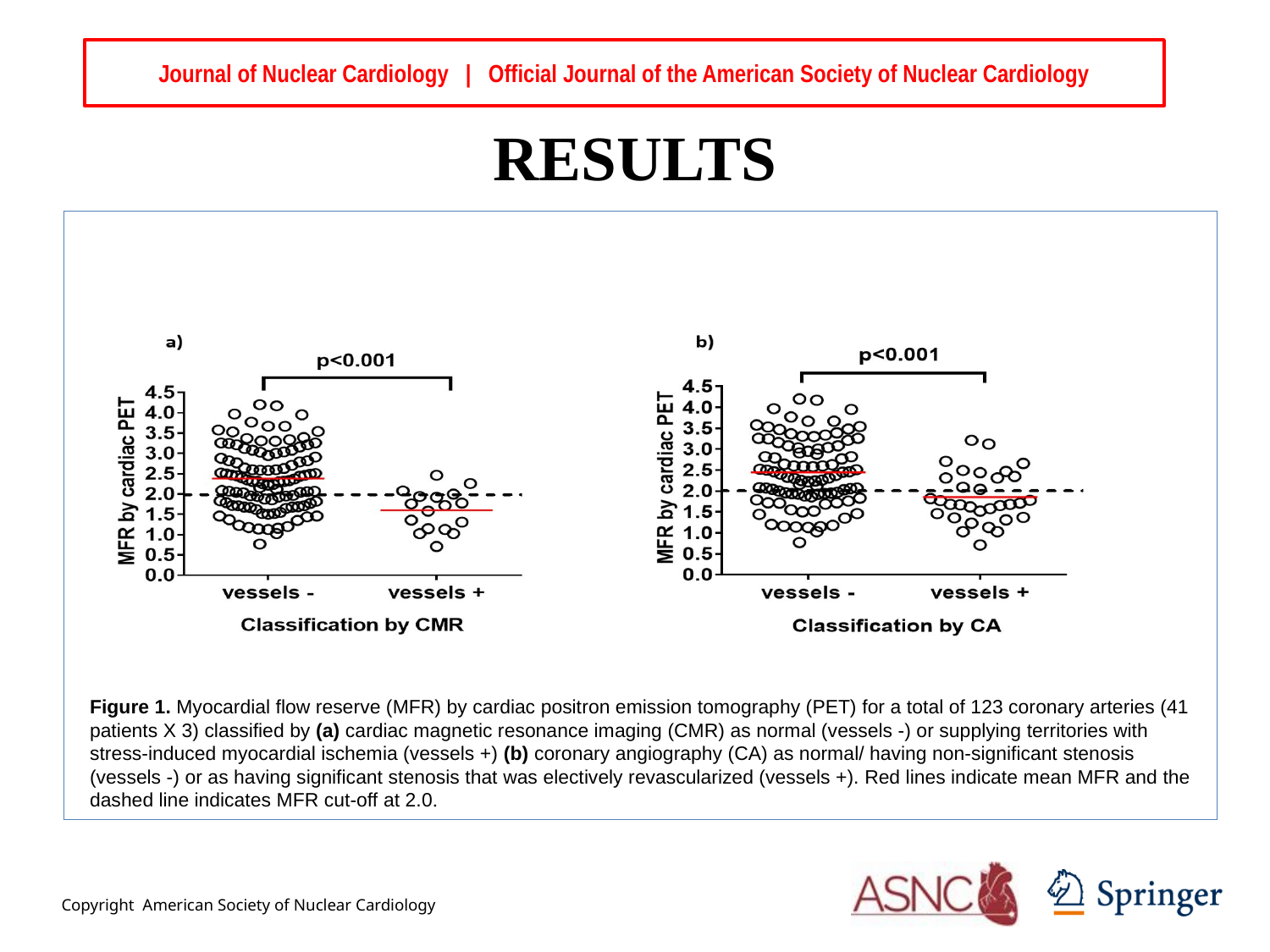

Journal of Nuclear Cardiology | Official Journal of the American Society of Nuclear Cardiology
# RESULTS
Figure 1. Myocardial flow reserve (MFR) by cardiac positron emission tomography (PET) for a total of 123 coronary arteries (41 patients X 3) classified by (a) cardiac magnetic resonance imaging (CMR) as normal (vessels -) or supplying territories with stress-induced myocardial ischemia (vessels +) (b) coronary angiography (CA) as normal/ having non-significant stenosis (vessels -) or as having significant stenosis that was electively revascularized (vessels +). Red lines indicate mean MFR and the dashed line indicates MFR cut-off at 2.0.
Copyright American Society of Nuclear Cardiology

## Slide 5
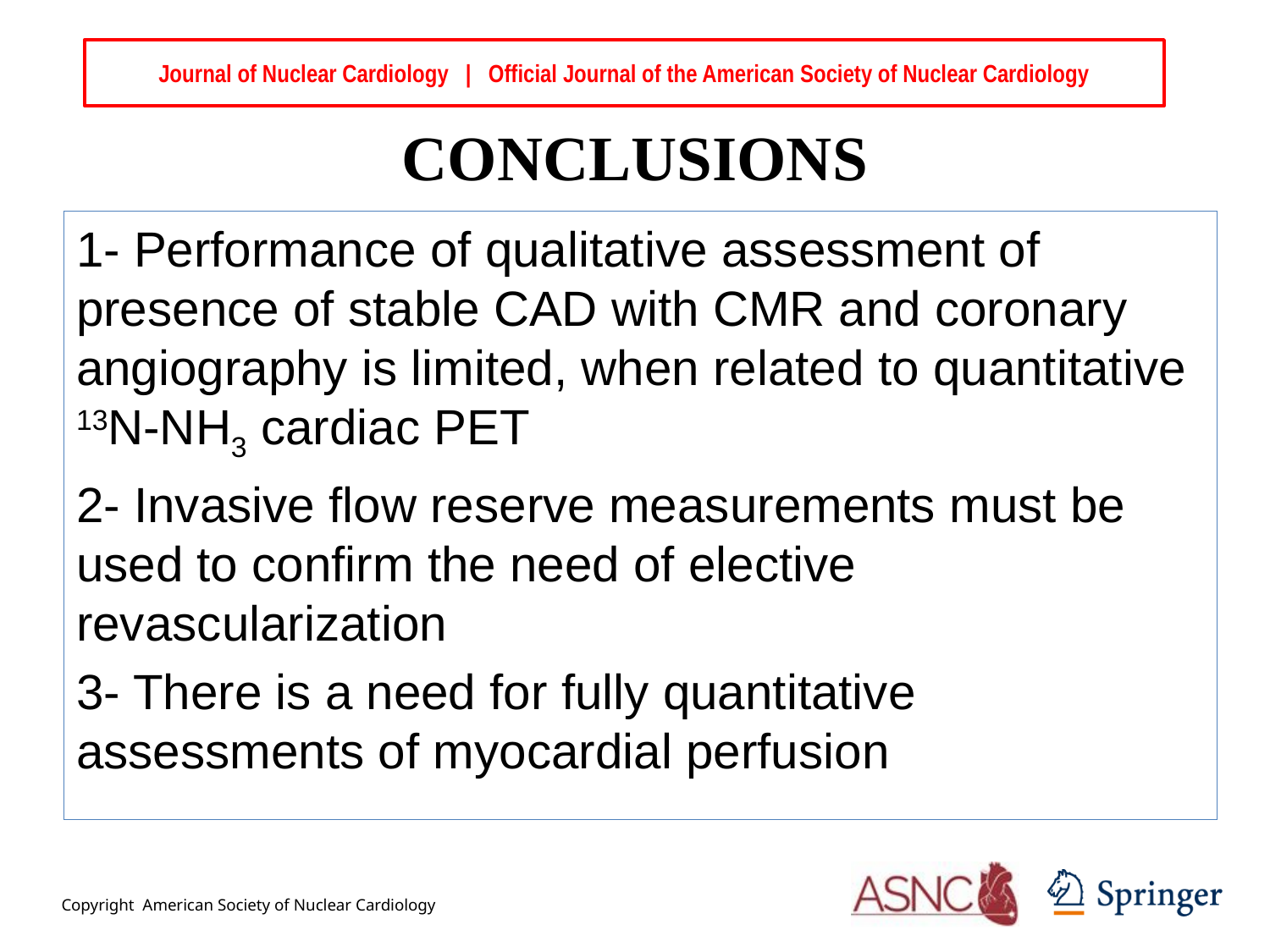

Journal of Nuclear Cardiology | Official Journal of the American Society of Nuclear Cardiology
# CONCLUSIONS
1- Performance of qualitative assessment of presence of stable CAD with CMR and coronary angiography is limited, when related to quantitative 13N-NH3 cardiac PET
2- Invasive flow reserve measurements must be used to confirm the need of elective revascularization
3- There is a need for fully quantitative assessments of myocardial perfusion
Copyright American Society of Nuclear Cardiology
